# Exogenous H_2_S Attenuates Hypertension by Regulating Renin Exocytosis under Hyperglycaemic and Hyperlipidaemic Conditions

**DOI:** 10.3390/ijms24021690

**Published:** 2023-01-14

**Authors:** Ning Liu, Mingyu Li, Siyuan Liu, Jiaxin Kang, Lingxue Chen, Jiayi Huang, Yan Wang, He Chen, Weihua Zhang

**Affiliations:** 1 Department of Pathophysiology, Harbin Medical University, Harbin 150086, China; 102464@hrbmu.edu.cn (N.L.); 2020020108@hrbmu.edu.cn (M.L.); jiaxinkang@hrbmu.edu.cn (J.K.); 2020020111@hrbmu.edu.cn (L.C.); 2018173029@hrbmu.edu.cn (J.H.); 2 Department of Urologic Surgery, First Affiliated Hospital of Harbin Medical University, Harbin 150001, China; siyuanliu@hrbmu.edu.cn (S.L.); yymnwangyan@126.com (Y.W.);; 3 Department of Forensic Medicine, Harbin Medical University, Harbin 150086, China

**Keywords:** type 2 diabetes mellitus, hypertension, H_2_S, renin release, autophagy

## Abstract

Obesity, along with type 2 diabetes mellitus (T2DM), is a major contributor to hypertension. The renin-angiotensin-aldosterone system is involved in the occurrence of diabetes and hypertension. However, the mechanism by which obesity is related to T2DM induced hypertension is unclear. In this study, we observed that blood pressure and serum renin content were increased in patients with diabetes and hypertension. Hydrogen sulfide (H_2_S), as an endogenous bioactive molecule, has been shown to be a vasodilator. Db/db mice, characterized by obesity and T2DM, and juxtaglomerular (JG) cells, which line the afferent arterioles at the entrance of the glomeruli to produce renin, treated with glucose, palmitic acid (PA) and oleic acid (OA), were used as animal and cellular models. NaHS, the H_2_S donor, was administered to db/db mice through intraperitoneal injection. NaHS significantly alleviated blood pressure in db/db mice, decreased the renin content in the serum of db/db mice and reduced renin secretion from JG cells. NaHS modulated renin release via cAMP and soluble N-ethylmaleimide-sensitive factor attachment protein receptors (SNAREs), including synaptosome-associated protein 23 (SNAP23) and vesicle-associated membrane protein 2 (VAMP2), which mediate renin exocytosis. Furthermore, NaHS increased the levels of autophagy-related proteins and colocalization with EGFP-LC3 puncta with renin-containing granules and VAMP2 to consume excessive renin to maintain intracellular homeostasis. Therefore, exogenous H_2_S attenuates renin release and promotes renin-vesicular autophagy to relieve diabetes-induced hypertension.

## 1. Introduction

Obesity, physical inactivity and energy-dense diets have led to a rapid growth in the population with type 2 diabetes mellitus (T2DM) [1]. Obesity is closely associated with T2DM and hypertension [2]. It has been estimated that approximately 75% of patients with T2DM suffer from hypertension [3]. Hypertension is a risk factor for diabetes-related vascular complications, such as myocardial infarction, stroke, congestive heart failure, and retinopathy [4]. Growing evidence has revealed that the renin-angiotensin-aldosterone system (RAAS) is involved in the occurrence of diabetes-induced hypertension [5,6]. The main rate-limiting step in the regulation of RAAS activity is the release of active renin from juxtaglomerular (JG) cells in the kidney [7,8]. The exocytosis of renin granules is stimulated by cAMP and SNARE (soluble N-ethylmaleimide-sensitive factor attachment protein receptor) family members [9,10,11,12].

Autophagy is a key molecular pathway that maintains cellular and organismal homeostasis [13,14]. Macroautophagy, the major type of autophagy, is a process that sequesters various cellular contents, such as proteins, lipids and damaged organelles within double-membraned autophagosomes. The autophagosomes then mature through fusion with lysosomes to form autolysosomes in which the packaged contents are degraded [15,16,17].

Hydrogen sulfide (H_2_S) is considered a gas transmitter that plays a protective role in multiple organ systems [18,19,20]. H_2_S attenuates oxidative stress, systemic inflammation, Ang II-induced hypertension, and spontaneous hypertension [21,22,23,24,25,26,27]. H_2_S induces vasorelaxation in the vasculature and contributes to the regulation of vascular tone to regulate blood pressure [22,28]. Our previous study demonstrated that H_2_S regulates autophagy through multiple pathways in diabetic cardiomyopathy [29,30]. The aims of this study were to investigate whether H_2_S regulates renin release by promoting the autophagy of renin vesicles and to decipher the mechanism by which the synaptosome-associated protein 23 (SNAP23) /vesicle-associated membrane protein 2 (VAMP2) pathway is inactivated under diabetic and hypertensive conditions.

## 2. Results

### 2.1. Blood Pressure of Diabetic Patients and db/db Mice

**Figure 1:**
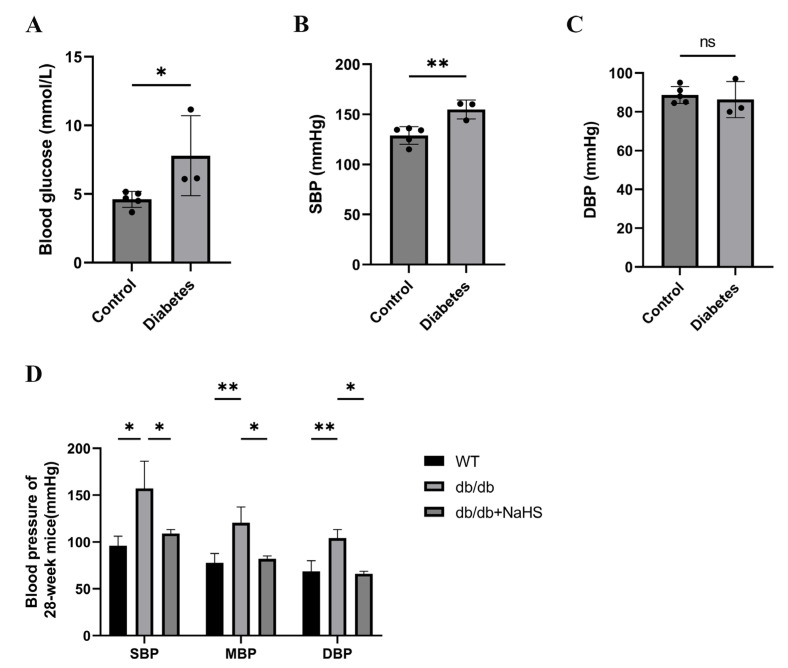
Blood pressure was measured in patients with diabetes and db/db mice. (**A**–**C**) Blood glucose, SBP and DBP of normoglycemic patients and patients with diabetes and hypertension (*n* = 3–5). (**D**) SBP, MBP and DBP in 28-week db/db mice (*n* = 4). * *p* < 0.05, ** *p* < 0.01. SBP, systolic blood pressure. MBP, mean blood pressure. DBP, diastolic blood pressure.

We investigated the occurrence of hypertension in patients with diabetes. The blood glucose and systolic blood pressure (SBP) of diabetes patients were significantly higher than those of normoglycemic patients, but there was no significant change in diastolic blood pressure (DBP) (Figure 1A–C). SBP, mean blood pressure (MBP), and DBP of 28-weeks old mice were assessed by the tail-cuff method to detect the occurrence of hypertension in db/db mice. These parameters were all increased in db/db mice and were obviously higher than those of the wild-type control group (Figure 1D). To observe the effect of exogenous H_2_S on blood pressure in db/db mice, an NaHS solution was injected intraperitoneally for 20 weeks beginning at 8 weeks. Our results showed that SBP, DBP and MBP in the db/db+NaHS group were lower than those in the db/db group (Figure 1D). These results implied that exogenous H_2_S might be involved in the regulation of blood pressure in type 2 diabetic mice.

### 2.2. Alteration of CSE Expression and H_2_S Levels in the Kidney Tissues of Diabetic Patients, db/db Mice and JG Cells

**Figure 2:**
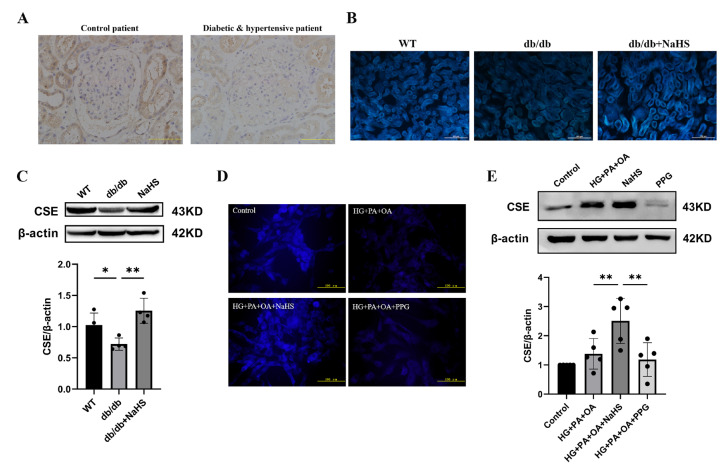
CSE expression and H_2_S levels were analyzed in the kidney tissues of diabetic patients, db/db mice and JG cells. (**A**) CSE expression in the kidney tissues of patients obtained during renal tumor surgery detected by immunohistochemistry. Scale bar = 50 μm. H_2_S content in the kidney tissues of db/db mice (scale bar = 500 μm) (**B**) and JG cells (**D**) was detected by C-7Az fluorescence probe. (**C**,**E**) Western blot analysis of CSE expression in the kidney tissues of db/db mice (*n* = 4) and in JG cells (*n* = 5) treated with NaHS (100 μM) and PPG (10 μM). * *p* < 0.05, ** *p* < 0.01. PA, palmitic acid. OA, oleic acid. JG, juxtaglomerular. CSE, cystathionine-γ-lyase. PPG, DL-propargylglycine.

To observe the alteration of H_2_S content in diabetes with hypertension, we assessed the expression level of CSE (cystathionine-γ-lyase), an H_2_S-producing enzyme, and the content of H_2_S in kidney tissues and JG cells. The immunohistochemistry staining results showed that the expression level of CSE in the kidney tissues of patients with diabetes and hypertension was decreased compared with that in normoglycemic patients (Figure 2A). JG cells were treated with 40 mM glucose, 400 μM palmitic acid (PA) and 200 μM oleic acid (OA) as a cellular model to mimic T2DM. A similarly altered pattern of CSE expression was consistently observed in db/db mice and JG cells as in patients with diabetes and hypertension. An H_2_S fluorescence probe (C-7A_Z_) was used to determine the H_2_S content [29,30,31]. The results revealed that NaHS obviously upregulated the CSE expression level and H_2_S content in db/db mice and JG cells (Figure 2B–E). Compared to the HG + PA + OA + NaHS groups, both the H_2_S content and CSE expression level were decreased in JG cells treated with the CSE inhibitor PPG (DL-propargylglycine) (Figure 2D,E). These results indicated that exogenous H_2_S could increase the H_2_S content and H_2_S production enzyme expression in the kidney tissues of db/db mice and JG cells under hyperglycaemic and hyperlipidaemic conditions.

### 2.3. The Effect of Exogenous H_2_S on Renin Expression, Renin Activity and Renin Release

**Figure 3:**
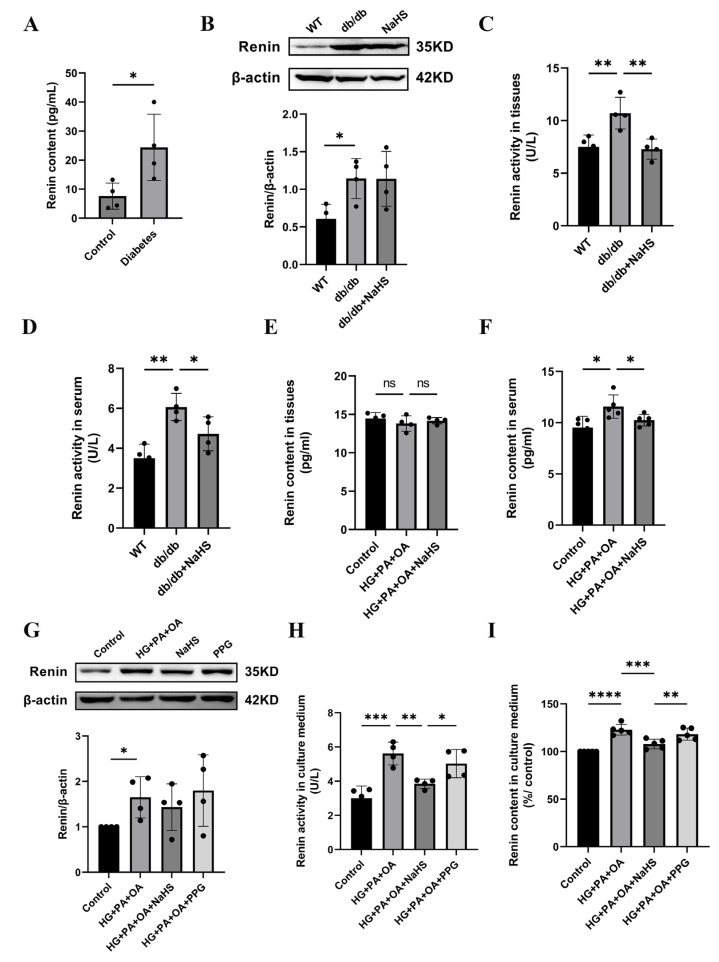
Effect of exogenous H_2_S on renin expression, renin activity and renin release. (**A**) Renin content in the serum of normoglycemic patients and patients with diabetes and hypertension (*n* = 4). (**B**) Renin expression level in kidney tissues of db/db mice was detected by Western blotting (*n* = 4). Renin activity and content in the serum and kidney tissues (**C**–**F**) and culture medium of JG cells (**H**,**I**) detected by ELISA kits (*n* = 4–5). (**G**) Western blot analysis of renin in JG cells (*n* = 4). * *p* < 0.05, ** *p* < 0.01, *** *p* < 0.001, **** *p* < 0.0001.

Previous studies have shown that the activation of the RAAS is involved in hypertension formation and that renin is a key factor in RAAS activation [32]. We found that the concentration of renin in serum was increased in patients with diabetes and hypertension (Figure 3A). To investigate whether H_2_S regulates blood pressure by mediating renin, we assessed the expression level of renin. Western blotting analysis showed that renin expression in kidney tissues of db/db mice was increased compared with that in the wild-type control group, while exogenous H_2_S did not reduce renin expression in db/db mice (Figure 3B). In a previous study, NaHS significantly decreased renin activity in the two-kidney-one-clip (2K1C) model of renovascular hypertension [33]. To investigate whether the increase in blood pressure in db/db mice was induced by RAAS activation, renin activity in kidney tissues and serum of db/db mice was determined using an ELISA kit. These results showed that the renin activity of the db/db group was significantly higher than that of the wild-type control group in both serum and kidney tissues, while the renin activity was significantly decreased after treatment with exogenous H_2_S (Figure 3C,D). Compared with the wild-type control group, the renin content in the kidney tissues of db/db mice was not significantly changed, while the renin content in serum was obviously elevated. These results indicate that exogenous H_2_S had no effect on renin content in kidney tissues but lessened the renin content in serum (Figure 3E,F). Renin is secreted by JG cells, and similar results in JG cells were observed as those in db/db mice. Renin expression was increased in the HG + PA + OA group; however, there was no alteration in the NaHS treatment group (Figure 3G). Compared with the control group, the renin activity and content in the culture medium were increased under hyperglycaemic and hyperlipidaemic conditions and lowered in the NaHS group (Figure 3H,I). Taken together, these results indicated that renin was activated and released into the circulation to activate the RAAS and participated in the occurrence of hypertension in db/db mice, while, exogenous H_2_S could reduce the activity, content and release of renin, thus inhibiting RAAS activation.

### 2.4. Exogenous H_2_S Inhibited RAAS Activation by Modulating the PDE3B-cAMP Pathway

**Figure 4:**
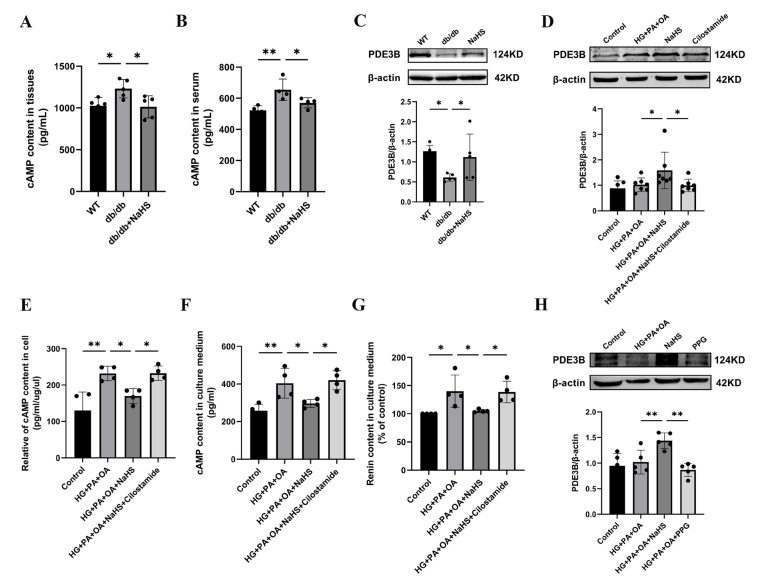
Exogenous H_2_S inhibited RAAS activation by modulating the PDE3B-cAMP pathway. (**A**,**B**) cAMP content in kidney tissues (*n* = 5) and serum of db/db mice (*n* = 4) assessed by an ELISA kit. (**C**,**D**) PDE3B expression levels in the kidney tissues (*n* = 5) and JG cells (*n* = 7) were detected by Western blotting. After treatment with cilostamide (10 μM) in JG cells, PDE3B expression level was detected. cAMP content in the intracellular (**E**) and cell culture medium (**F**), and renin content in cell culture medium (**G**) were assessed by ELISA kits (*n* = 4). (**H**) Western blot analysis of PDE3B expression level in JG cells treated with PPG (*n* = 5). * *p* < 0.05, ** *p* < 0.01. PDE3B, phosphodiesterases 3B.

Renin release is regulated by the cAMP/PKA signaling pathway [9]. To further investigate how H_2_S regulates renin release, the content of cAMP was determined using an ELISA kit. As shown in Figure 4A,B, the cAMP content in the kidney tissues and serum was elevated in the db/db group and decreased in the db/db+NaHS group. Phosphodiesterase (PDE) plays a role in hydrolyzing cAMP [7,34]. To investigate whether PDE3B regulates cAMP content in db/db mice, PDE3B expression levels were measured by Western blot analysis. The protein level of PDE3B was decreased in kidney tissues of db/db mice and JG cells under hyperglycaemic and hyperlipidaemic conditions, or in JG cells treated with the PDE3B inhibitor, cilostamide. Exogenous H_2_S significantly increased PDE3B expression (Figure 4C,D). To examine the effect of cAMP on renin release, the contents of cAMP and renin were determined. The results showed that exogenous H_2_S decreased cAMP content and renin content in the culture medium of JG cells, which were reversed by treatment with cilostamide (Figure 4E–G). To further explore the effect of H_2_S on renin release, JG cells were treated with PPG. The results indicated that PPG downregulated PDE3B expression level and increased the renin content in the cell culture medium (Figure 3I and Figure 4H). These data demonstrated that exogenous H_2_S could reduce cAMP content and then inhibit renin release.

### 2.5. Exogenous H_2_S Attenuated Renin Release via SNARE Proteins

**Figure 5:**
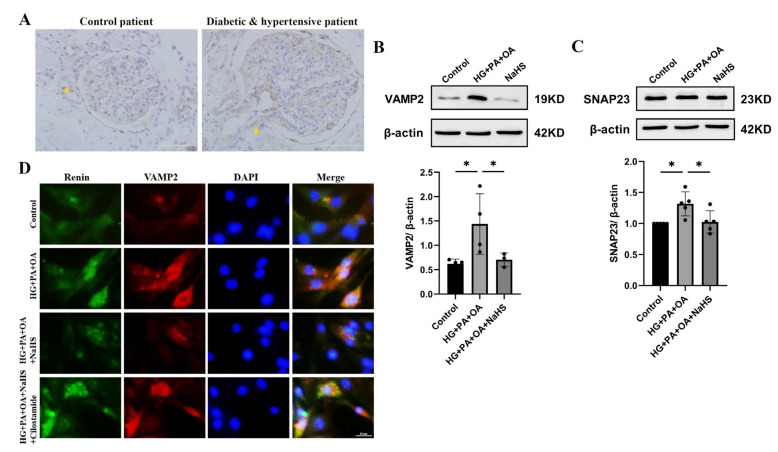
Exogenous H_2_S attenuated renin release via SNARE proteins. (**A**) Immunohischemistry of the expression of VAMP2 in kidney tissues of patients. Scale bar = 50 μm. (**B**,**C**) VAMP2 and SNAP23 expression levels in JG cells were measured by Western blotting (*n* = 5). (**D**) The colocalization of renin with VAMP2 in JG cells was detected by immunofluorescence staining. Scale bar = 20 μm. * *p* < 0.05. VAMP2, vesicle-associated membrane protein 2. SNAP23, synaptosome-associated protein 23.

SNARE proteins are involved in the process of cAMP-stimulated renin release and exocytosis [10,11,12]. To investigate whether exogenous H_2_S affected renin release by regulating SNARE proteins, the expression levels of VAMP2 and SNAP23 were determined by immunohistochemistry staining and Western blot analysis. The experimental results showed that the protein level of VAMP2 was elevated in the kidney tissues of patients with diabetes and hypertension (Figure 5A). In addition, the expression levels of VAMP2 and SNAP23 were increased in JG cells under hyperglycaemic and hyperlipidaemic conditions, and NaHS lowered the expression levels (Figure 5B,C). To further assess the effect of SNARE proteins on renin release, the interaction between renin-containing granules and VAMP2 was determined by immunofluorescence staining. Exogenous H_2_S reduced the colocalization of renin-containing granules with VAMP2. To explore whether H_2_S enhances the exocytosis of renin mediated by cAMP, JG cells were treated with cilostamide. As shown in Figure 5D, cilostamide increased the colocalization of renin with VAMP2 compared with the NaHS-treated group. These results implied that exogenous H_2_S decreased the exocytosis of renin by attenuating vesicle transportation.

### 2.6. Exogenous H_2_S Promoted Renin Consumption through Activation of Autophagy

**Figure 6:**
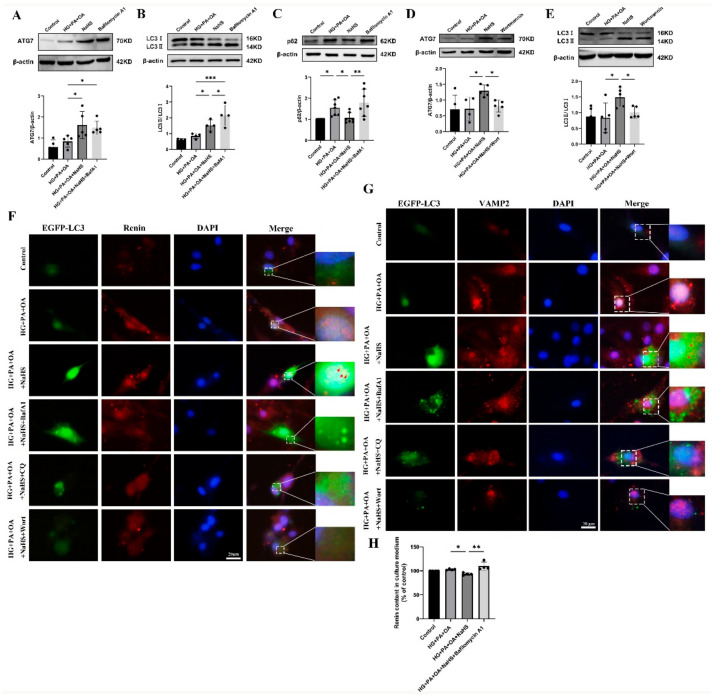
Exogenous H_2_S promoted renin consumption through activation of autophagy. (**A**–**E**) The expression levels of ATG7, LC3II/I and p62 were detected by Western blotting after treatment with bafilomycin A1 (100 nM) or wortmannin (1 μM) (*n* = 4–7). (**F**,**G**) The colocalization of renin-containing granules with autophagosomes in JG cells detected by immunofluorescence staining after transfection with EGFP-LC3 plasmid. Scale bar = 20 μm, (**H**) After treatment with an autophagy inhibitor, the renin content in the cell culture medium of JG cells was detected using ELISA kits. * *p* < 0.05, ** *p* < 0.01, *** *p* < 0.001. BafA1, bafilomycin A1. CQ, chloroquine. Wort, wortmannin.

Macroautophagy can sequester intracellular cargo into autophagosomes, which are finally degraded by binding with lysosomes [14,15,16,17]. To determine whether H_2_S regulates autophagy in JG cells under hyperglycaemic and hyperlipidaemic conditions, western blotting was used to assess the expression levels of autophagy-related proteins. Western blotting analysis showed that exogenous H_2_S increased the expression levels of ATG7 and LC3 while decreasing the expression level of p62 (Figure S1). Bafilomycin A1 (BafA1), a vacuolar V-ATPase inhibitor, prevents the fusion of autophagosomes with lysosomes and inhibits lysosomal degradation [35]. After treatment with bafilomycin A1, the protein levels of ATG7, LC3 and p62 were all increased (Figure 6A–C). However, the protein levels of ATG7 and LC3 were decreased after treatment with the PI3K inhibitor wortmannin (wort) (Figure 6D,E), which can inhibit autophagosome formation [36]. These results indicated that exogenous H_2_S promoted the formation and degradation of autophagosomes.

To further explore whether H_2_S regulated the degradation of excess renin protein through autophagy, JG cells were transfected with the EGFP-LC3 plasmid. The colocalization of autophagosomes with VAMP2 and renin was increased in the NaHS group, as determined by immunofluorescence staining (Figure 6F,G), suggesting that exogenous H_2_S upregulated the fusion of renin-containing granules with autophagosomes. In addition, the number of EGFP-LC3 puncta was increased in the bafilomycin A1- or lysosomal inhibitor chloroquine (CQ)-treated group and decreased in the wortmannin-treated group. To further show that the effect of autophagy on renin release is regulated by H_2_S, the renin content in the culture medium of JG cells was determined. Exogenous H_2_S decreased the renin content, which was increased after treatment with bafilomycin A1 (Figure 6H). Taken together, these results suggested that exogenous H_2_S promoted the degradation of renin vesicles by regulating autophagy to reduce renin release.

## 3. Discussion

In a previous study, H_2_S was shown to regulate blood pressure and play a protective role in multiple organ systems [18,19,20,22,26]. Our results showed that blood pressure was higher in diabetic patients and db/db mice than in normoglycemic groups. The expression of CSE was decreased, and renin content was elevated in the serum of patients with diabetes and hypertension, in the serum of db/db mice, and in the cell culture medium of JG cells under hyperglycaemic and hyperlipidaemic conditions. We propose that exogenous H_2_S attenuates the exocytosis of renin and promotes the degradation of renin vesicles, thereby lowering blood pressure.

H_2_S plays a role as an endothelial cell-derived relaxing factor through activation of ATP-sensitive potassium (K_ATP_) channels directly, leading to vascular smooth muscle cells relaxation and serving as a vasodilator [37,38]. In addition, H_2_S reduced blood pressure in a 2K1C model of renovascular hypertension, and NaHS reduced Ang II-induced hypertension and spontaneous hypertension [26,33]. A previous study suggested that deficiency in H_2_S-generating enzymes is a key factor that predisposes patients to the induction of hypertension [39,40]. In this study, we found that blood pressure was decreased after treatment with exogenous H_2_S in db/db mice. Thus, H_2_S enhancement may be an alternative approach for treating diabetes with hypertension.

Diabetes and hypertension have similar mechanisms, including increased oxidative stress and RAAS activation [41]. The main rate-limiting step in the regulation of RAAS activity is the release of active renin from JG cells. Previous research has shown that the exocytosis of renin-containing granules participates in renin release, which is activated by the cAMP/PKA pathway [9]. Cyclic nucleotide phosphodiesterases (PDEs) are a class of phosphohydrolytic enzymes that selectively catalyze the hydrolysis of 3- cyclic adenosine phosphate and/or 3-, 5-cyclic guanosine phosphate [7]. PDE3 and PDE4, which are expressed in renal vessels and JG cells, can degrade cAMP. PDE3 contains two subtypes, PDE3A and PDE3B. In *Pde3b*-deficient mice, energy homeostasis regulation is altered, and metabolic disorders occur (including systemic insulin resistance) [42]. In this study, we found that exogenous H_2_S inhibited renin release by decreasing cAMP content through the regulation of PDE3B. These results were consistent with previous research showing that H_2_S inhibits the activity of adenylate cyclase in the myocardium and smooth muscle cells and reduces the production of cAMP, thereby inhibiting renin release. We found that exogenous H_2_S decreased cAMP content by increasing PDE3B expression in db/db mice and JG cells under hyperglycaemic and hyperlipidaemic conditions, and the PDE3B inhibitor cilostamide increased the renin content in culture medium compared with NaHS. These results indicated that exogenous H_2_S regulated renin release by mediating cAMP.

Accumulating evidence indicates that the SNARE protein is involved in vesicle transport [43]. Once secreting cells are stimulated, to mediate fusion, at least 3 SNARE family members, including a vesicle-associated membrane protein (VAMP), a syntaxin and one type of synaptosome-associated protein (SNAP), interact to form a stable four-helix complex through their coiled-coil domains [10]. In the kidney, specific SNARE isoforms are expressed, including VAMP2 and VAMP3, syntaxin 3 and 4, and SNAP23. Previous studies have shown that VAMP2 is a subtype of VAMP that must interact with SNAP to mediate renin release stimulated by cAMP via exocytosis in JG cells [12]. A previous study reported that SNAP23 is highly expressed in the renal cortex and exists in JG cells to participate in the regulatory release of renin [10]. In our study, VAMP2 expression was increased in the kidney tissues of diabetic patients with hypertension. Compared with the HG + PA + OA group, NaHS decreased the expression levels of VAMP2 and SNAP23 and reduced the colocalization of renin-containing granules with VAMP2 in JG cells. However, cilostamide had the opposite effect as NaHS. Collectively, these results indicated that H_2_S might regulate renin exocytosis and release via a SNARE protein-cAMP mechanism. The potential mechanism will be elucidated in the future studies, including how H_2_S regulates SNARE proteins.

In this study, we found that there was no obvious change in renin content in the kidney tissues between the NaHS group and db/db group, but the renin content was increased in the serum of db/db mice compared with NaHS supplementation. Our previous study found that exogenous H_2_S regulated autophagy in diabetic cardiomyopathy [29,30]. We hypothesized that renin might be consumed after supplementation with exogenous H_2_S. Currently, three forms of autophagy have been identified, including macroautophagy, chaperone-mediated autophagy, and microautophagy. Macroautophagy is a highly conserved autophagic degradation pathway that maintains homeostasis in eukaryotes. We found that NaHS increased the expression levels of the autophagy-related proteins ATG7 and LC3II and decreased p62. Surprisingly, we found that the number of EGFP-LC3 puncta and the colocalization of EGFP-LC3 puncta with renin granules both increased in the NaHS group. These data supported the hypothesis that the excess renin protein within JG cells was degraded through autophagy regulated by exogenous H_2_S to maintain intracellular homeostasis.

## 4. Materials and Methods

### 4.1. Study Participants

We selected normoglycemic patients and patients with diabetes and hypertension for the study. Hyperglycemia is characterized by fasting glucose ≥7.8 mmol/L, or taking antidiabetic agents for T2DM treatment. Hypertension is characterized by systolic blood pressure (SBP) ≥140 mmHg and/or diastolic blood pressure (DBP) ≥ 90 mmHg, or taking general antihypertensive agents.

Ten individuals underwent renal tumor surgery after the assessment of surgical eligibility at the Department of Urology, the First Affiliated Hospital of Harbin Medical University. Clinical and laboratory data for each patient were collected within one week before their surgery. The patients in the study were in a supine position, and fasting blood (after overnight fasting for 10–12 h) was collected in the early morning (8:00–10:00 a.m.). Blood glucose, levels of serum renin and blood pressure were detected. Kidney biopsies of patients were obtained during the urology surgery, fixed with 4% paraformaldehyde, and stored at 4 °C.

All patients involved in this study provided written informed consent. This study was approved by the Institutional Review Board of Harbin Medical University.

### 4.2. Animal Studies

The animal experiments in this study were performed and in accordance with the Guide for the Care and Use of Laboratory Animals published by the China National Institute of Health and approved by the Animal Care Committees of Harbin Medical University, China.

Homozygous male and female mice with leptin receptor deficiency (db/db mice) that can develop obesity and T2DM were used in this experiment. Six-to seven-week-old- db/db mice (*n* = 50) were kept in the animal experimental center of Harbin Medical University for 1–2 weeks. The relevant wild-type mice (*n* = 30) were used as the control group. Blood glucose was measured and db/db mice with blood glucose over 16.7 mmol/L were randomly divided into two groups. NaHS (H_2_S donor, 40 μmol/kg/2 d) solution was administered to db/db mice through intraperitoneal injection as in the db/db+NaHS group for 20 weeks. The tissues and related experiments were derived from 28-week-old mice.

### 4.3. Blood Pressure Measurement of Mice

Twenty-eight-weeks old mice were subjected to a noninvasive tail-cuff method to measure the blood pressure of the mice as previously described [44]. Then, they were adjusted to a stable condition for 10 min in a quiet and warm environment. The Softron bp-2010 mouse blood pressure monitor was opened and connected. The mice were put into the mouse net, then into the heat preservation tube, and then into the rat bag. The pressure receptor was placed at the tail root of the mouse, and the tip of the pressure receptor marker was in the same direction as the tip of the mouse tail. SBP, DBP and MBP were measured. Blood pressure values were recorded and averaged.

### 4.4. Serum and Tissue Collection

After feeding for 20 weeks, mice were fasted for 12 h and anesthetized with 3% pentobarbital sodium solution (50 mg/kg) through intraperitoneal injection. Blood samples were collected in a blood collection tube and centrifuged for 10 min at 3000 r/min. The supernatant was taken as serum. For each group, approximately 1/2 of the kidney tissues of mice were placed in 4% paraformaldehyde and fixed for frozen sections. The remaining kidney tissues and other mouse tissues were stored at −80 °C after rapid freezing with liquid nitrogen for subsequent experiments.

### 4.5. Immunohistochemistry

Small pieces of kidney tissues were obtained from diabetic and hypertensive patients or normoglycemic patients during renal tumor surgery. The kidney tissues were fixed in 4% paraformaldehyde, paraffin sectioned, deparaffinized, rehydrated, antigen repaired, blocked, and incubated with CSE and VAMP2 overnight at 4 °C. The sections were incubated with secondary antibody at 37 °C for 1 h and treated with nerve HRP tracer chromogenic solution (DAB method, avoiding light). The nuclei were counterstained with hematoxylin for 1 min, washed with tap water, and dehydrated with an alcohol gradient. The sections were cleared with xylene solution, and observed under a microscope after sealing with neutral resin.

### 4.6. Culture, Treatment and Transfection of JG Cells

DMEM (Gibco, Thermo Fisher Scientific, MA, USA, 4.5 g/L D-glucose, L-glutamine, 110 mg/L sodium bicarbonate, 10% FBS, 1% penicillin and streptomycin) was used to culture JG cells. After being passaged, cells were confluent at approximately 60% and then treated with 40 mM glucose, 400 μm palmitate (PA) and 200 μm oleate (OA) for 48 h as a cellular model to mimic T2DM for subsequent experiments.

EGFP-LC3 plasmid (Addgene, Watertown, MA, USA, plasmid 11546) was transfected into JG cells usingLipofectamin 3000 (Thermo Fisher Scientific, Waltham, MA, USA) according to the manufacturer’s instruction.

### 4.7. Immunofluorescence Staining

After treatment of mouse kidney tissues by sucrose gradient dehydration in 4% paraformaldehyde, the tissue blocks were covered with OCT embedding agent, and then frozen sections were made using the freezing section machine, with a thickness of 5–10 μm. Triton X-100 (0.1%) was used for membrane penetration for 10–20 min, and then 3% BSA solution was used to block the sections or JG cells for 30 min. Anti-renin antibody (Proteintech, Wuhan, China) and anti-VAMP2 antibody (Proteintech) were used and incubated overnight at 4 °C. The sections or JG cells were incubated with TRITC-labeled or FITC-labeled fluorescent secondary antibody at 37 °C for 1.5–2 h (kept in a dark humid box). Then, the nuclei were stained with DAPI and placed at 25 °C for 5–10 min. The expression of renin was observed under a fluorescence microscope.

### 4.8. Measurement of H_2_S Level

To detectH_2_S levels, the H_2_S probe 7-azoid-4-methylcoumarin (C-7Az, 50 μmol/L, Sigma-Aldrich, St. Louis, MO, USA) was used to stain the frozen sections of kidney tissues and JG cells. The samples were stained at 37 °C away from light for 30 min. After washing with PBS three times, H_2_S level (fluorescence intensity) was observed through fluorescence microscopy.

### 4.9. Western Blot Analysis

The cytoplasmic proteins of JG cells or kidney tissues were obtained and lysed in cell lysis buffer with phenylmethylsulfonyl fluoride (PMSF). The BCA method was used to measure the protein concentration. SDS-PAGE was performed to separate the protein samples which were then transferred to NT nitrocellulose membranes (Pall Corporation, Beijing, China), blocked in blocking buffer (diluted in TBST) and incubated with the primary antibody. The membranes were incubated in horseradish peroxidase (HRP)-conjugated secondary antibodies for 1.5–2 h at 25 °C. The luminescent signal was visualized using ECL plus luminescent image analyzer. The results were analyzed using ImageJ, and anti-β-actin was used as an internal reference.

The primary antibodies were purchased as follows: Renin (Proteintech), CSE (Proteintech), PDE3B (Proteintech), SNAP23 (Proteintech), VAMP2 (Proteintech), LC3 (Proteintech), ATG7 (Proteintech), p62 (Proteintech), and β-actin (Proteintech).

### 4.10. Measurement of Renin Activity and Content

Renin activity and content in serum, kidney tissues and culture medium of JG cells were detected using an enzyme-linked immunosorbent assay kit (Jiang Lai, Shanghai, China). Briefly, a small part of kidney tissues (all groups were uniform quality) was cut and the corresponding volume of PBS (containing PMSF) was added. The tissues were homogenized sufficiently on the ice. The homogenate was then centrifuged at 5000× *g* (4 °C condition) for 5–10 min, and the supernatants were assessed. The culture medium of JG cells was collected. Then, the renin activity and content were assessed according to the manufacturer’s directions.

#### 4.11. cAMP Content Measurement

The cAMP levels in serum, kidney tissues and culture medium of JG cells were detected using an ELISA kit (Mei Mian, Wuhan, China) according to the manufacturer’s directions. The samples were described as above.

### 4.12. Statistical Analysis

All results of this experiment were statistically analyzed using Prism 9.4.1 (GraphPad, La Jolla, CA, USA) software. All data are expressed as the mean ± standard deviation. In this study, one-way ANOVA was used for comparison among multiple groups, and an independent sample *t* test was used for data comparisons between two groups. *p* < 0.05 indicates a significant statistical difference.

## 5. Conclusions

In summary, this study provided evidence that exogenous H_2_S mediated cAMP and SNARE proteins to regulate renin exocytosis and promoted the consumption of excessive renin granules via autophagy, therefore attenuating the blood pressure of db/db mice. This work may provide new insights and potential therapeutic targets of H_2_S for the treatment of type 2 diabetes with hypertension.

## Data Availability

All data and materials are available upon request.

## Supplementary Materials

The following supporting information can be downloaded at: https://www.mdpi.com/1422-0067/24/2/1690/s1.
