# Correction: Liu et al. Exogenous H_2_S Attenuates Hypertension by Regulating Renin Exocytosis under Hyperglycaemic and Hyperlipidaemic Conditions. *Int. J. Mol. Sci.* 2023, *24*, 1690

**DOI:** 10.3390/ijms24086996

**Published:** 2023-04-10

**Authors:** Ning Liu, Mingyu Li, Siyuan Liu, Jiaxin Kang, Lingxue Chen, Jiayi Huang, Yan Wang, He Chen, Weihua Zhang

**Affiliations:** 1 Department of Pathophysiology, Harbin Medical University, Harbin 150086, China; 2 Department of Urologic Surgery, First Affiliated Hospital of Harbin Medical University, Harbin 150001, China; 3 Department of Forensic Medicine, Harbin Medical University, Harbin 150086, China

In the original publication [1], there was a mistake in Figure 6. We revised the Western blotting band of Figure 6B, which was uploaded incorrectly, and the groups in the bar charts of Figure 6A–E, which were marked incorrectly by accident. The corrected Figure 6 appears below.

There were two figure numbers that were writing mistakes in Sections 2.3 and 2.4. The authors have replaced “there was no alteration in the NaHS treatment group (Figure 3H)” with “there was no alteration in the NaHS treatment group (Figure 3G)” in Section 2.3. And the authors replaced “Figures 3J and 4H” with “Figures 3I and 4H” in Section 2.4.

The authors apologize for any inconvenience caused and state that the scientific conclusions are unaffected. This correction was approved by the Academic Editor. The original article has been updated.

## Figures and Tables

**Figure 6:**
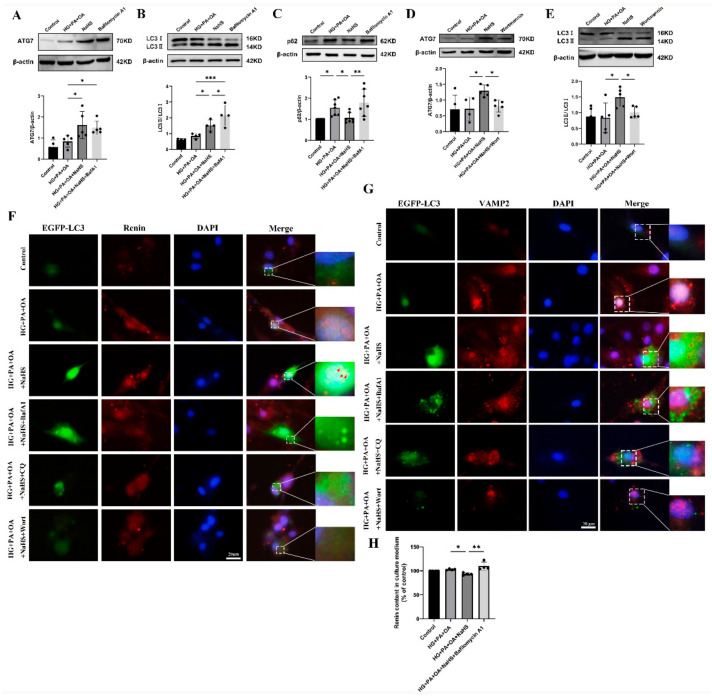
Exogenous H_2_S promoted renin consumption through activation of autophagy. (**A**–**E**) The expression levels of ATG7, LC3II/I and p62 were detected by Western blotting after treatment with bafilomycin A1 (100 nM) or wortmannin (1 μM) (*n* = 4–7). (**F**,**G**) The colocalization of renin-containing granules with autophagosomes in JG cells detected by immunofluorescence staining after transfection with EGFP-LC3 plasmid. Scale bar = 20 μm, (**H**) After treatment with an autophagy inhibitor, the renin content in the cell culture medium of JG cells was detected using ELISA kits. * *p* < 0.05, ** *p* < 0.01, *** *p* < 0.001. BafA1, bafilomycin A1. CQ, chloroquine. Wort, wortmannin.
