# Supplementary material for: Exogenous H2S Attenuates Hypertension by Regulating Renin Exocytosis under Hyperglycaemic and Hyperlipidaemic Conditions
Source: Int J Mol Sci. 2023 Jan 14;24(2):1690. doi: 10.3390/ijms24021690 (PMC9860892; doi:10.3390/ijms24021690)
Supplement: Supplementary file 1 [file ijms-24-01690-s001.zip › ijms-2077324-supplementary.pdf]

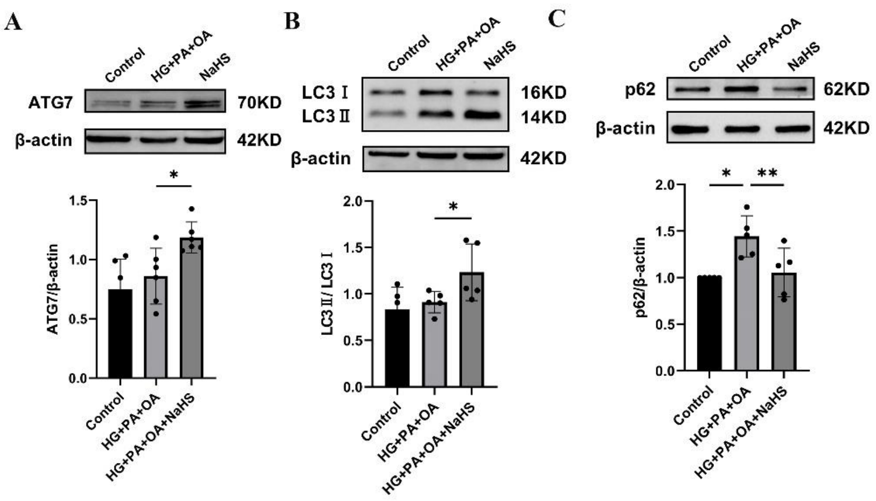

Figure S1. Exogenous H_2S regulated autophagy-related proteins expression. The expression levels of ATG7 **(A)**, LC3 Ⅱ/Ⅰ **(B)** and p62 **(C)** were detected by western blotting. * p <0.05, ** p <0.01, n=5-6.
